# The Importance of Moral Construal: Moral versus Non-Moral Construal Elicits Faster, More Extreme, Universal Evaluations of the Same Actions

**DOI:** 10.1371/journal.pone.0048693

**Published:** 2012-11-28

**Authors:** Jay J. Van Bavel, Dominic J. Packer, Ingrid Johnsen Haas, William A. Cunningham

**Affiliations:** 1 New York University, New York, New York, United States of America; 2 Lehigh University, Bethlehem, Pennsylvania, United States of America; 3 Department of Political Science, University of Nebraska-Lincoln, Lincoln, Nebraska, United States of America; 4 The University of Toronto, The Ohio State University, Toronto, Ontario, Canada; University College London, United Kingdom

## Abstract

Over the past decade, intuitionist models of morality have challenged the view that moral reasoning is the sole or even primary means by which moral judgments are made. Rather, intuitionist models posit that certain situations automatically elicit moral intuitions, which guide moral judgments. We present three experiments showing that evaluations are also susceptible to the influence of moral versus non-moral construal. We had participants make moral evaluations (rating whether actions were morally good or bad) or non-moral evaluations (rating whether actions were pragmatically or hedonically good or bad) of a wide variety of actions. As predicted, moral evaluations were faster, more extreme, and more strongly associated with universal prescriptions—the belief that absolutely nobody or everybody should engage in an action—than non-moral (pragmatic or hedonic) evaluations of the same actions. Further, we show that people are capable of flexibly shifting from moral to non-moral evaluations on a trial-by-trial basis. Taken together, these experiments provide evidence that moral versus non-moral construal has an important influence on evaluation and suggests that effects of construal are highly flexible. We discuss the implications of these experiments for models of moral judgment and decision-making.

## Introduction

Over the past decade, intuitionist models of morality have challenged the view that moral reasoning is the sole or even primary means by which moral judgments are made. Rather, intuitionist models posit that certain situations automatically elicit moral intuitions, which guide moral judgments [1]. According to these models, moral judgments are very often produced by reflexive mental computations that are unconscious, fast, and automatic [2]. From this perspective, affective responses are automatically triggered by certain moral issues and provide a strong bottom-up influence on judgments and decision-making. As such, the role of moral reasoning is relegated to the role of *post hoc* justification [1] or corrective control following the initial intuition [3], but is not the causal impetus for a moral judgment. In the current paper, we present three experiments showing that moral evaluations are also susceptible to construal. Specifically, we show that people can deliberately construe a wide variety of actions through either a moral or a non-moral lens with different consequences for their evaluations.

### The Origins of Moral Intuitions

Dating back to Darwin [4], several theorists have proposed that evolution may have provided humans with a built-in set of moral rules, heuristics or intuitions [5], [6], [7], [8], [9]. In addition, moral beliefs and values can develop through social learning, via which children learn specific cultural practices [1], [10] and ultimately acquire a set of knowledge structures about moral standards that guide their social interactions and provide the foundation for morality in adulthood [11]. The conversion of preferences into values—termed moralization—often occurs in cultures and individuals on the scale of years and involves an increased overlap between values and a personally or socially important issue or action [12], [13].

The work on the biological and cultural basis of morality has inspired a highly influential approach to moral psychology—the intuitionist model. The intuitionist model of moral judgment focuses on evaluations “that are made with respect to a set of virtues held to be obligatory by a culture or subculture” [1] This definition is broad enough to allow “marginally moral judgments” that may have escaped the attention of moral philosophers but are nevertheless moralized in the local cultural milieu (e.g., eating a low fat diet). Whereas, rationalist approaches hold that moral judgments are reached through a process of reasoning and reflection [14], [15], [16], the intuitionist approach argues that eliciting situations automatically trigger affective moral intuitions, which guide moral judgments [1]. According to the intuitionist model, conscious reasoning frequently follows an initial judgment, providing a *post hoc* justification but not the causal impetus.

For instance, Haidt and colleagues [17] have created scenarios to which people typically have strong moral reactions but fail to articulate any rational principles to justify their responses—a response termed ‘moral dumbfounding’. Likewise, there is now extensive experimental evidence that disgust and other emotional responses influence moral judgments [18], [19]. From the intuitionist perspective, unconscious, affective responses guide reactions to these morally charged scenarios and people often engage in deliberate reasoning only after they have already made an initial moral judgment.

Despite the popularity of the bottom-up approach to morality posited by the intuitionist model, several theorists have argued that an appraisal process [20] is a pre-requisite for generating specific emotional intuitions [9], [21]. The fact that cultural shifts and individual differences in moralization occur suggests that morality is not always intrinsic to stimuli, but may be the result of construing those actions as morally-relevant [22]. Moreover, there is evidence that different people construe different issues in moral (or non-moral) terms—termed moral mandates [23]. It is unclear, however, whether individual differences in the moralization of specific issues is based on intuitions built on biological and cultural differences or construal processes. This raises a critical question: can people quickly change between a moral versus non-moral construal of the same action or issue?

### A Dynamic Model of Evaluation

Although it may seem sensible that individuals can appraise or construe the same action in different ways, there is a surprising lack of empirical evidence on this issue in the domain of morality. In a recent paper on the dynamic nature of evaluation, we hypothesized that evaluation should indeed be sensitive to moral versus non-moral construal processes [24]. For example, people may be able to construe a situation or stimulus in moral or non-moral terms depending on their goals and beliefs, which will direct attention, modulate perception and guide consequent emotional intuitions. For the purposes of the present research, we use the terms moral and non-moral to describe different evaluative modes. This over-simplified distinction reflects the fact that participants are explicitly told to make moral evaluations (how right or wrong is an action) in each study. To provide a contrast with moral evaluations, participants also make pragmatic (how personally good or bad is an action) or hedonic (how personally pleasant or unpleasant is an action) evaluations. These latter conditions are termed “non-moral” simply because participants are not explicitly asked to make moral evaluations. We are aware that certain participants may consider hedonic maximization or self-interest moral imperatives [25], [26]. Indeed, Kohlberg [27] considered self-interest the second stage of moral reasoning.

To test our hypothesis, in the current research we directly manipulated the way people evaluated a wide variety of actions to determine whether construal has an influence on evaluations of the exact same actions.

Our predictions are grounded in a dynamical model of evaluation—termed the Iterative Reprocessing (IR) Model [24], [28], [29]. Whereas many dual-process models characterize human evaluation as a function of automatically activated associations and subsequent, corrective control processes [30], the IR Model highlights the dynamic interactions between multiple component processes in the evaluative system. A key assumption underlying our model is that brain systems are organized hierarchically, such that lower–order automatic processes influence *and* are influenced by higher–order processes [31]. As such, reflective processes do not merely override or control automatic ones—these processes work in a dynamic, interactive fashion to construct evaluations. In this way, object construal plays an important role in determining evaluations, including shaping the initial response to a stimulus.

The IR Model makes a distinction between the *contents* (e.g., attitudes and representations), *processes* (e.g., mental operations and computations) and *outcomes* of evaluation [28]. Thus, while people may develop relatively stable moral *content* (e.g., standards and values), whether these contents influence an *evaluation* at any given moment likely depends on whether an action or issue is processed in moral or non-moral terms (in this paper, we use the term “processed” in the broad sense to include stimulus construal). Although it is likely the case that highly moralized actions (like murder) are chronically and reflexively *processed* as moral (i.e., the representations rapidly stabilize in a way that reflects the moral construal) and are therefore commonly evaluated in moral terms, we propose that many actions can be evaluated according to moral considerations [28]. A helpful analogy is available in the social psychology literature. When perceivers categorize targets as in-group members it has important implications for their perceptions, evaluations and behavior [32], [33], and can even override ostensibly automatic biases to visually salient categories like race [32], [34], [35]. Thus, construal or categorization can even shape automatic evaluations of stimuli with strong affective associations [36].

In the current research, we test the prediction that a wide variety of actions can be evaluated using both moral and non-moral considerations, and that this construal process can lead to different evaluative outcomes for the same actions. In three experiments, we instructed people to evaluate the same stimuli in moral versus non-moral (i.e., pragmatic or hedonic) terms. By holding the influence of the stimuli constant while varying the construal, we were able to investigate the influence of moral versus non-moral construal. This ensures that differences observed in the nature of evaluative outcomes are due to differences in the construal (or an interaction between construal and stimuli) rather than the mere influence of the stimuli. If our assumptions are accurate, evaluating actions on the basis of moral versus non-moral considerations should lead to different evaluative outcomes. As long as there is some moral *content* that participants can bring to bear on their evaluation, moral construal may alter the evaluation of actions that have not been typically seen as moral. For example, one can bring to mind the moral aspects of recycling (e.g., saving the environment) even if more pragmatic aspects normally predominate (e.g., the time and effort involved). Of course, there may be some actions that have virtually no moral content to draw upon when generating an evaluation. For those stimuli, moral and non-moral evaluative outcomes may be similar.

### The Flexibility of Moral Construal

We are not the first to suggest that people can flexibly construe and evaluate the same actions as moral or not [37]. For instance, models of ethical decision-making distinguish between moral *awareness*, in which a person recognizes that a situation may have moral relevance, and moral *judgment*, in which the moral value of a course of action is determined. These models predict that only if a person is morally aware will they apply processes to render a moral judgment [38], [39], [40]. The division between these stages is important because it can account for particular types of moral failure in which people make immoral decisions not because they intended to do so or because they mistakenly evaluate an immoral act as moral, but rather because they fail to consider the action on the basis of moral considerations in the first place. Although moral awareness and moral judgment are conceptually distinct, the majority of psychological research on morality has focused on the latter, investigating how characteristics of the perceiver, the stimulus and/or the social context affect judgments of right or wrong regarding issues that are ostensibly morally-relevant [18], [19], [41], [42]. However, before making a moral judgment, the evaluative system must be ready to evaluate the action in moral terms—people have to construe the stimulus as potentially morally relevant.

In related work, Tetlock and colleagues [43] have proposed that people are multifunctional entities who shift between different decision-making frameworks depending on the context and their current goals. Thus, the same person may alternate between acting as an “intuitive economist” animated by utilitarian goals, and a “principled theologian” animated by the need to protect sacred values from secular encroachments. To investigate the tension between these forms of evaluation, they forced participants to consider tradeoffs between moral and pragmatic values—termed a taboo trade-off. As it turns out, people often react with moral outrage when a material valuation is placed on sacred items or events [44]. This research highlights that sometimes an opposition exists between moral and pragmatic evaluative processes, and that when pitted against one another, moral considerations typically dominate judgments. Again, however, this opposition may be unique to specific types of highly moralized stimuli; further, these studies require participants to engage in pragmatic and moral forms of evaluation simultaneously. To better understand the differences between moral and non-moral evaluation, the current research separates and compares moral and non-moral construals of the same actions.

Some recent research suggests that moral judgments are not intrinsic to issues, but are the result of construing actions as morally-relevant (i.e., moral awareness). In one paper, directing participants' attention to an action that violates moral rules elicited *deontological* preferences, whereas directing their attention to the outcomes that favored the violation of a moral rule elicited *utilitarian* preferences [21]. In a different paper, participants were randomly assigned to rate 70 stimuli—including a subset of 20 mundane objects (e.g., refrigerator, desk)—on how *morally good or bad* they thought the stimuli were or how much they *liked or disliked* the stimuli [45]. Although the mean ratings were not directly compared across these two conditions, the mundane objects were judged positively (relative to the mid-point of the scale) in the moral condition. These studies not only suggest that people can view relatively mundane stimuli as having moral value, but that construal can change the evaluation [46].

### The Current Research

We present three experiments in which participants were instructed to evaluate the same stimuli—a wide range of positive and negative actions—in moral and/or non-moral terms. In each of the experiments, we asked participants to make moral and non-moral evaluations of the same actions to determine whether moral *(rating whether actions were morally good or bad)* relative to non-moral *(rating whether actions were pragmatically or hedonically good or bad)* construal would lead to different evaluative outcomes. We assumed that potential courses of action could be construed in multiple ways, and that *how* they were construed would influence the nature of the evaluations. By holding stimuli constant, we could examine whether moral versus non-moral construal can influence evaluations. If we observe differences in the nature of resulting evaluations and associated judgments it would provide evidence that the distinction between moral construal (which is triggered by a situational cue in this case) and evaluation stages is an important one, and that moral and non-moral modes of evaluation can be flexibly applied to the same stimuli.

To examine the influence of moral construal, we employed a task facilitation paradigm [47]. This paradigm allowed us to determine whether making a moral versus non-moral evaluation about one's actions was associated with universality judgments about the behavior of others. The paradigm was based on the following logic: if the process of performing the first task (i.e., generating an evaluation) or the information activated during the first task was relevant to the second task (i.e., making a universality judgment), then the time needed to perform the second task should be reduced [48]. Therefore, to assess the extent to which two or more tasks rely on similar processes/information, one can analyze the degree to which performing the first task diminishes the time needed to complete the second task. The task facilitation effect will be greatest when the processes or information are highly similar in both tasks. Similarly, any differences in task facilitation between conditions will reflect the differential relevance of processes or activated information rather than differences in stimuli (which were held constant). It is also possible that differences between conditions might reflect aspects of task interference rather than facilitation.

In the current research, the first task involved a moral, pragmatic or hedonic evaluation and the second task was a universality judgment. We compared the average reaction time for universality judgments between conditions to determine whether these judgments were more strongly associated with moral, pragmatic or hedonic evaluative modes. We chose to examine universality judgments because universality is widely considered to be a hallmark of moral cognition. Moral philosophers and psychologists have long posited that moral evaluations are (or should be) associated with universal prescriptions—the belief that absolutely everybody should act in the same way [49], [50], [51], [52], [53]. Other psychologists have argued that moral attitudes are experienced as matters of fact that others could or should be persuaded to share, rather than as matters of preference, taste or convention [16], [23], [54]. Further, compared to conventional transgressions, for example, moral transgressions are consistently rated as more wrong, punishable, independent of authority, and universally applicable. These differences emerge early in life and appear to hold across societies [55], [56], [57]. We predicted that if moral evaluation is more strongly linked to universality than other forms of evaluation, the time required to make a universality judgment should be shorter following a moral evaluation than a non-moral evaluation. We also assessed whether moral evaluations were more highly correlated with the subsequent universality judgments, and predicted that this correlation should be stronger than the correlation between pragmatic evaluations and the subsequent universality judgments.

In addition, we hypothesized that construing actions in different ways would give rise to observable differences in evaluation, despite holding the stimuli constant. Empirical work suggests that moral evaluation entails black-and-white thinking and moral absolutes. For example, moral attitudes are more durable and resistant to temptation [13], and are associated with stronger reactions to dissimilar others [23], both of which are indicators of attitude strength [58]. As noted, other research suggests that moral judgments are often based on moral intuitions or heuristics [1], leading to quick and simple judgments. We therefore predicted that moral evaluations would be more extreme and rendered faster than non-moral evaluations of the same actions. However, research on moral reasoning raised the alternative prediction that moral evaluations might be more deliberate and, therefore, take longer than non-moral evaluations [59]. Our paradigm allowed us to directly test these competing hypotheses by comparing participants' reaction times to moral and non-moral evaluations of the same stimuli.

We also sought to examine whether people could shift back-and-forth between moral and non-moral evaluations of the same objects. Although studies have recently suggested that moral awareness may be relatively flexible [45], none have directly examined whether or not people are able to shift back-and-forth between moral and non-moral construals of the same stimuli within the same session. Our multi-level model of the human evaluative system assumes that top-down influences on evaluation are highly flexible and update rapidly [24], [28], [29]. We therefore anticipated that people could evaluate actions in moral or non-moral terms in a flexible fashion. To examine this possibility, we had participants switch back-and-forth between moral and non-moral evaluation. As elaborated above, we predicted that evaluations would be faster, more extreme, and more strongly associated with universally prescriptive judgments following moral as compared with pragmatic or hedonic evaluations—and that these effects would shift to reflect the current moral versus non-moral evaluative mode, even if these shifts were separated by mere seconds.

## Experiment 1

### Overview and Predictions

In the first experiment, participants made moral and pragmatic evaluations of a wide variety of actions—including actions typically construed in moral terms (e.g., murder, honesty), and actions that are not (e.g., riding a bike, eating). In order to test whether moral evaluations were more universally prescriptive than pragmatic evaluations, after rating each action in moral or pragmatic terms, participants then rated how many other people should/should not engage in the action (universality judgment). Each trial consisted of an evaluation (moral or pragmatic) followed by a universality judgment of the same action. We measured the ratings and reaction time for the evaluation and the universality judgment. In addition to exploring the relationship between different evaluations and universality, we used this information to test whether moral (relative to pragmatic) evaluations were associated with faster and more extreme evaluations.

We predicted that if moral evaluations are more strongly linked to universality than pragmatic evaluations, two things should occur. First, the time required to make a universality judgment should be shorter following a moral evaluation than a pragmatic evaluation. Second, moral evaluations should be highly correlated with the subsequent universality judgments, and this correlation should be stronger than the correlation between pragmatic evaluations and the subsequent universality judgments.

### Material and Methods

#### Participants

Forty-five undergraduate students (26 females; mean age = 20 years) participated for partial course credit for an Introduction to Psychology course. One participant was removed from analysis for failing to follow instructions.

#### Procedure

Participants arrived at the lab in small groups and completed all tasks on individual computers. Participants read that they would be presented with a number of different behaviors (e.g., getting a flu shot) and would be asked to evaluate them. They were also told that there were at least two ways of evaluating an action: “One way of evaluating an action is by thinking about whether it would be good or bad for you personally. These pragmatic judgments focus on pros and cons, and take into account the benefit or the harm *you* may experience if you do something. A second way of evaluating an action is by thinking about how moral or ethical it is. Rather than thinking about what would benefit you personally, these moral judgments focus on whether or not you ought to do something because it is the right or the wrong thing to do.” Participants were also told that after evaluating each action, they would be asked to rate how many other people should engage in that behavior.

**Figure 1:**
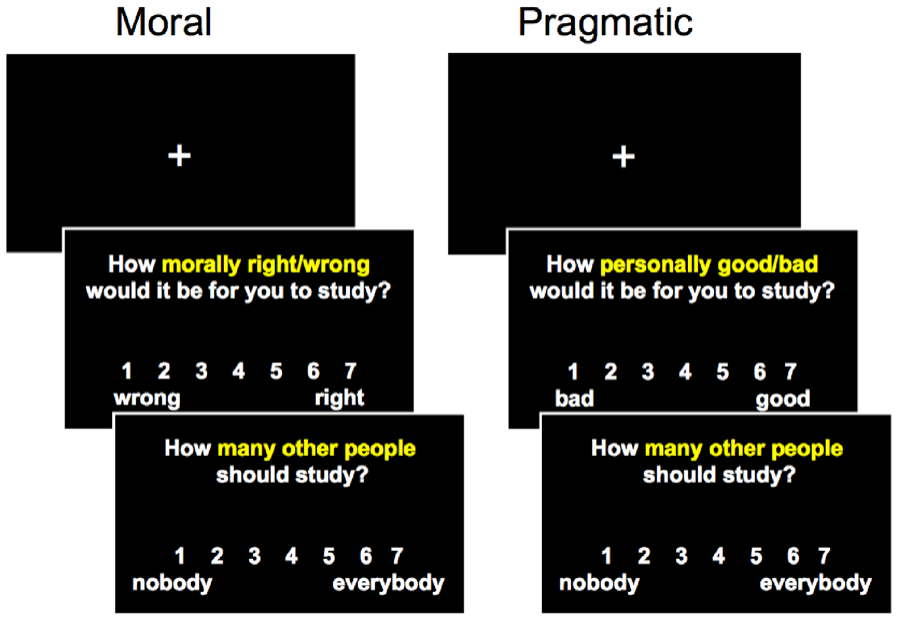
A visual representation of the moral and pragmatic evaluation trials presented in Experiment 1. On each trial, a fixation cross appeared for 1,000 ms before participants made a moral or pragmatic evaluation followed by a universality judgment. We recorded reaction times on the moral/pragmatic evaluation and university judgment. The trials were presented in four blocks. In each block, participants made moral and universality evaluations for 13 actions before switching to pragmatic and universality evaluations for 13 different actions.

Participants were presented with 104 actions (e.g., recycle, shop-lift, study; see Appendix S1A for complete list of stimuli) one at a time on a desktop computer using E-Prime (see Figure 1). Participants made *moral evaluations* for 52 actions using the keyboard, rating “how morally wrong/right it would be for you to [action]” (1 = *very wrong* to 7 = *very right*), and *pragmatic evaluations* for the other 52 actions, rating “how personally bad/good you think it would be for you to [action]” (1 = *very bad* to 7 = *very good*). Actions remained on screen until participants made a response (M = 3,683 ms). Following each moral and pragmatic judgment, participants made *universality judgments* for the same action, rating “how many other people should [action]” (1 = *nobody* to 7 = *everybody*).

The actions were presented in four blocks. In each block, participants made moral and universality evaluations for 13 actions before switching to pragmatic and universality evaluations for 13 different actions. The order of moral and pragmatic judgments was counterbalanced such that half of the participants made moral judgments first within each block, and half made pragmatic judgments first. Actions were randomly assigned within participants to be evaluated morally versus pragmatically. Participants never made a moral and pragmatic evaluation of the same action; however, across participants, each action was equally likely to be evaluated according to moral or pragmatic standards. This ensured that any differences between moral and pragmatic evaluations were not due to the specific actions but to differences in moral versus pragmatic evaluation.

#### Analyses

To assess differences between moral versus pragmatic evaluation, we conducted 2 (evaluation type: moral, pragmatic)×4 (block: 1, 2, 3, 4) analyses of variance (evaluation type and block were repeated measure factors) on the speed with which participants made evaluations, the overall valence and extremity of their evaluations, and their reaction times to subsequent universality ratings. To analyze reaction times, we removed trials with extremely slow (>10,000 ms) reaction times and log-transformed all remaining reaction times to minimize the influence of outliers and skewness [60]. To ease interpretation, all reported means are based on raw reaction times. Analyses with raw and log-transformed reaction times were nearly identical.

Traditional analyses of repeated measures have tended to focus on mean-level differences in reaction time or accuracy. However, this approach has the consequence of reducing hundred of trials to a single score for each participant diminishing power and meaningful variance. To more accurately measure moral and pragmatic judgments we used multi-level modeling [61]. Multi-level modeling allows for the direct analysis of accuracy on individual trials and helps overcome violations of independence that occur as a result of correlated trials within participants. When an assumption of independence is not satisfied, ignoring dependency among trials can lead to invalid statistical conclusions; namely the underestimation of standard errors and the overestimation of the significance of predictors [62]. We therefore created multi-level models with trials nested within participants to provide more appropriate estimates of regression parameters. Multi-level models were implemented in the SAS PROC MIXED procedure [63].

### Results

#### Moral evaluations are associated with universality

Our primary prediction was that moral evaluations would be more strongly associated with universality judgments than pragmatic evaluations. To test this hypothesis, we compared the reaction times of universality judgments following moral versus pragmatic evaluations. As predicted, participants were faster to make universality judgments following moral (M = 1,254 ms) compared to pragmatic (M = 1,443 ms) evaluations, *F*(1, 43) = 9.17, *p*<.01. As shown in Table 1, participants were also faster to make universality judgments during later blocks (a practice effect), *F*(3, 129) = 72.46, *p*<.01; however, the effect of condition was not moderated by block (*p* = .94). Moreover, an item-by-item analysis indicated that evaluating an action in moral terms facilitated subsequent universality judgments regardless of the moral rating it received—even actions that were rated as morally neutral led to faster universality judgments. These results demonstrate that moral evaluations facilitated universality judgments more than pragmatic evaluations throughout the study, suggesting that participants were able to switch between moral and pragmatic evaluative modes.

**Table 1:** Mean responses following moral versus pragmatic evaluations in Experiment 1.

DV	Task	Block 1	Block 2	Block 3	Block 4	Average
Evaluation RT	Moral	4,448	3,618	3,283	3,031	3,595 (148)
	Pragmatic	4,748	3,866	3,566	3,326	3,877 (148)
Extremity	Moral	5.82	5.81	5.80	5.96	5.85 (.19)
	Pragmatic	5.59	5.75	5.61	5.40	5.59 (.19)
Universality RT	Moral	1,806	1,247	1,071	893	1,254 (100)
	Pragmatic	2,116	1,384	1,255	1,016	1,443 (100)

Means are provided for raw reaction times (RT; in milliseconds) and extremity of responses for each block. Excludes all trials with reaction times >10,000 ms. Overall scores may not reflect mean Block scores due to rounding errors and missing trials. Pooled standard errors in parentheses.

To examine whether this facilitation effect held across the full range of actions or was specific to actions with certain moral ratings we conducted an item-level analysis. We calculated means across participants for each action: its mean moral rating, and separate mean reaction times for universality judgments following moral and pragmatic evaluations. Using a hierarchical regression analysis, we then regressed mean reaction times for universality judgments on preceding evaluation type (moral vs. pragmatic), the mean moral rating of each action and their interaction term. Consistent with the primary analysis, there was a significant main effect of preceding evaluation type, such that participants were faster to make universality judgments following a moral than a pragmatic evaluation (*p*<.01). There were also linear and curvilinear effects of moral rating: actions with higher mean moral ratings (i.e., actions rated as more moral) were associated with slower universality judgments (*p* = .05) and actions with extreme moral ratings (i.e., highly immoral and moral actions) were associated with faster universality judgments (*p*<.01). Critically, the main effect of preceding evaluation type was not moderated by the linear (*p*>.60) or curvilinear (*p*>.90) moral rating terms.

We also examined whether universality judgments were more highly correlated with preceding moral than pragmatic evaluations. As predicted, a two-way interaction between evaluation type and the preceding moral/pragmatic rating, *F*(1, 43) = 11.18, *p*<.01, indicated that participants' universality ratings were more strongly associated with preceding moral (ß = .93) than pragmatic (ß = .83) ratings. As such, participants were more likely to indicate that nobody should engage in actions evaluated as immoral relative to actions evaluated as personally negative; conversely, participants were more likely to indicate that everybody should engage in actions evaluated as moral relative to actions evaluated as personally positive. Once again, this interaction was not moderated by block (*p* = .58). These results demonstrate that universality judgments (nobody/everybody) were more highly correlated with moral (wrong/right) than pragmatic (bad/good) ratings. In sum, these results are consistent with the general hypothesis that moral evaluations are associated with universality judgments to a greater degree than pragmatic evaluations.

#### Moral evaluations are extreme

We predicted that moral evaluations would be more positive and/or more extreme than pragmatic evaluations. Whereas previous research has suggested that people who rate objects on whether they are *morally good or bad* may come to rate them more positively [45], we found no difference on the overall ratings of actions when participants made moral (M = 4.07) or pragmatic (M = 4.04), *F*(1, 43) = .22, *p* = .64, and there was no interaction with block (*p* = .22).

**Figure 2:**
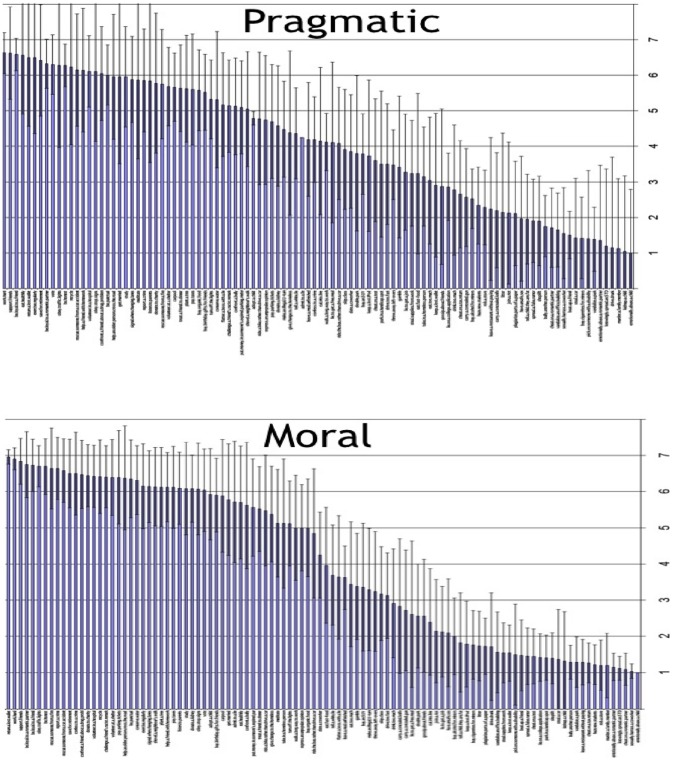
The mean pragmatic and moral ratings (with standard errors) for each action in Experiment 1. The actions have been rank ordered on the Y-axis from the highest (left) to lowest (right) mean rating. The X-axis reflects the rating scale (range 1–7). Pragmatic ratings are relatively linear whereas moral ratings are curvilinear, reflecting differences in extremity.

We predicted that moral evaluations would be more extreme than pragmatic evaluations of the same actions. To test this hypothesis, we computed and compared the extremity of moral versus pragmatic ratings. Since all moral/pragmatic ratings ranged in valence from one to seven, we created curvilinear extremity scores by mean centering and squaring each rating. For example, a rating of 5 (out of 7) would be computed by subtracting the overall mean (4.05) and squaring the difference (.95)*(.95) to provide an extremity score (.90). As shown in Table 1, participants made marginally more extreme moral (M = 5.85) than pragmatic (M = 5.59) ratings of the same actions, *F*(1, 43) = 3.54, *p* = .067. Consistent with the prediction that participants would be able to switch between moral and pragmatic evaluative modes, the effect of evaluation type was not moderated by block (*p* = .39). These results indicate that moral evaluations were more extreme than pragmatic evaluations of the same actions (see Figure 2).

#### Moral evaluations are fast

We predicted that moral judgments would be faster than pragmatic evaluations of the same actions. To test this hypothesis, we compared the reaction times of moral versus pragmatic evaluations. As predicted, participants were faster to provide moral (M = 3,595 ms) than pragmatic (M = 3,877 ms) evaluations of the same actions, *F*(1, 43) = 11.29, *p*<.01. As shown in Table 1, participants were faster to respond in later blocks, *F*(3, 129) = 99.86, *p*<.01, indicating a task-learning effect; however, the effect of condition was not moderated by block (*p* = .97). These results demonstrate that moral evaluations were faster than pragmatic evaluations throughout the study, suggesting that these were distinct modes of evaluation and participants were able to switch back and forth between moral and pragmatic evaluative modes.

### Discussion

Consistent with our predictions, moral and pragmatic construals of the same actions were associated with distinct evaluative outcomes. Moral evaluations made on the same set of actions were faster, more extreme and more universally prescriptive than pragmatic evaluations. Further, these distinct consequences were maintained as participants switched back-and-forth between moral and pragmatic evaluations, indicating that these evaluations are not only distinct, but are also highly sensitive to current top-down construal.

These findings are consistent with what is known about the flexibility of the human evaluative system [24], [28] and suggest that many issues may not necessitate automatic and inflexible construals. Although many issues, such as incest or pushing someone off of a footbridge, may evoke moral considerations, Experiment 1 suggests that people can deliberately construe and evaluate a host of issues in reference to moral considerations. Thus, while chronic moralization about many issues may elicit strong attitudes [1], [12], [23], construal can shape the evaluation of many of these same issues and lead to several different evaluative outcomes.

The results from Experiment 1 provide evidence that thinking morally is associated with universality. Specifically, participants were not only faster to make a universality judgment following a moral than a pragmatic evaluation, but mean moral judgments were more highly correlated with mean universality judgments than pragmatic judgments. However, the wording of the universality item was general enough that it could imply normativity or desirability. Classic research on morality has shown that it is important to distinguish moral norms from mere social conventions or personal preferences. Further, by asking where “how many other people should” engage in a given action we may have left open the definition of “other people”. Participants may interpret “other people” to mean group members at almost any level of social categorization (e.g., university students, Americans, humans). Consequently, narrow interpretations of “other people” allows for relativism, as any moral norm may only be applied to a narrow subset of humanity.

We were, however, interested in assessing the relationship between moral evaluation and universal moral duty—what Kant termed the “categorical imperative” [50]. Categorical imperatives are moral principles that are intrinsically valid and must be obeyed by all people in all situations and circumstances. According to Kant, people should “Act only according to that maxim whereby you can at the same time will that it should become a universal law” [50]. To better approximate this construct, participants in Experiment 2 were asked “whether each action should be universally prohibited or required, where universal means that something applies to all people, without limit or exception”.

The results from Experiment 1 indicated that moral evaluations were faster and more extreme than pragmatic evaluations. These results are consistent with scientific and lay understandings of morality. However, they may also be due to the difference in the scales used for moral versus pragmatic evaluations: pragmatic evaluations were made on a scale from *very bad* to *very good* whereas moral evaluations were made on a scale from *very wrong* to *very right*. Although both evaluations were made on 7-point scales, the different labels that anchored each scale may have led to different interpretations. One possibility is that the right/wrong anchors may have implied more extreme judgments during moral evaluation [64]. Further, the more extreme labels could have primed a specific mindset that facilitated subsequent universality judgments. Alternatively, the right/wrong anchors may have been interpreted to mean the normativity or correctness of an action (regardless of moral content). For example, participants may have made extreme judgments because some actions are simply correct (e.g., using keys to start a car) and others are incorrect (e.g., using keys to start a refrigerator). We addressed these concerns in the following experiments by holding the scales for moral and pragmatic types of evaluation constant—participants evaluated every action on a scale from *very bad* to *very good*.

## Experiment 2

### Overview and Predictions

In the second experiment, participants made moral and pragmatic evaluations of a wide variety of actions to determine whether moral evaluations were more strongly associated with universal prescriptions than pragmatic evaluations. In order to test this hypothesis, participants rated each action as moral or pragmatic and then rated whether the action should be universally prohibited/required. We also attempted to replicate the results from Experiment 1 showing that moral evaluations are associated with faster and more extreme evaluative outcomes than pragmatic evaluations while holding the scale labels constant for both types of evaluation.

### Material and Methods

#### Participants

Seventy undergraduate psychology students (50 females; mean age = 19) participated for partial course credit. Four participants were removed from analysis for failing to follow instructions.

#### Procedure

The procedure was similar to Experiment 1, with three important differences. The first difference was the inclusion of a different universality question. After evaluating each action, participants were asked whether the action should be universally prohibited or required, where universal means that something applies to all people, without limit or exception. Participants were told “For something to be universally prohibited it means that nobody should be permitted to do this action, without exception. For something to be universally required it means that everybody should be required to do this action, without exception.” Participants made these ratings on a 7-point scale (1 = *universally prohibited* to 7 = *universally required*). The second difference was holding the scale labels constant for moral and pragmatic evaluations. Specifically, participants made *moral evaluations* for 60 actions, rating “how morally bad/good it would be for you to [action]” (1 = *very bad* to 7 = *very good*), and *pragmatic evaluations* for the other 60 actions, rating “how personally bad/good you think it would be for you to [action]” (1 = *very bad* to 7 = *very good*). Following each moral and pragmatic evaluation, participants made *universality judgments* for the same action. The third difference was the inclusion of 16 additional actions during evaluation (see Appendix S1B; for a total of 120 actions).

Actions were presented in four blocks. In each block, participants made moral and universality evaluations for 13 actions before switching to pragmatic and universality evaluations for 13 different actions. The order of moral and pragmatic judgments was counterbalanced such that half of the participants made moral judgments first within each block, and half made pragmatic judgments first. Actions were randomly assigned within participants to be evaluated morally versus pragmatically. Participants never made a moral and pragmatic evaluation of the same action; however, across participants, each action was equally likely to be evaluated according to moral or pragmatic standards. This ensured that any differences between moral and pragmatic evaluations were not due to the specific actions.

### Results

To assess differences between moral versus pragmatic evaluation, we conducted 2 (evaluation type: moral, pragmatic)×4 (block: 1, 2, 3, 4) analyses of variance (where evaluation type and block were repeated measure factors) on the speed with which participants made evaluations, the overall valence and extremity of their evaluations, and their reaction times to subsequent universality ratings. To analyze reaction times, we removed trials with extremely long (>10,000 ms) reaction times and log-transformed all remaining reaction times. To ease interpretation, all reported means are based on raw reaction times.

#### Moral evaluations are associated with universality

Our primary prediction in Experiment 2 was that moral evaluations would be more strongly associated with universality judgments than pragmatic evaluations. To test this hypothesis, we compared the reaction times of universality judgments following moral versus pragmatic evaluations. As predicted, participants were faster to make universality judgments following moral (M = 1,438 ms) compared to pragmatic (M = 1,542 ms) ratings, *F*(1, 65) = 8.66, *p*<.01. A main effect of block indicated that participants were faster to make universality judgments during later blocks, *F*(3, 195) = 172.29, *p*<.01; however, the effect of evaluation type on the speed of universality judgments was not moderated by block (*p* = .50). Moreover, an item-by-item analysis indicated that evaluating an action in moral terms facilitated subsequent universality judgments regardless of the moral rating it received—even actions that were rated as morally neutral. Replicating the results from Experiment 1, moral evaluations facilitated universality judgments more than pragmatic evaluations throughout the study, suggesting that participants were able to switch between moral and pragmatic evaluative modes.

As in Experiment 1, we conducted an item-level analysis to examine whether this facilitation effect held across the full range of actions or was specific to actions with certain moral ratings. Consistent with the primary analysis, there was a significant main effect of preceding evaluation type, such that participants were faster to make universality judgments following a moral than a pragmatic evaluation (*p*<.03). There were also linear and curvilinear effects of moral rating: actions with higher mean moral ratings (i.e., actions rated as more moral) were associated with slower universality judgments (*p*<.02) and actions with extreme moral ratings (i.e., highly immoral and moral actions) were associated with faster universality judgments (*p*<.01). Critically, the main effect of preceding evaluation type was not moderated by the linear (*p*>.15) or curvilinear (*p*>.30) moral rating terms.

Following the results of Experiment 1, we predicted that universality judgments would be more highly correlated with preceding moral than pragmatic evaluations. As predicted, a two-way interaction between evaluation type and the preceding moral/pragmatic rating, *F*(1, 65) = 20.83, *p*<.01, indicated that participants' universality ratings were more strongly associated with preceding moral (ß = .75) than pragmatic (ß = .70) ratings. As such, participants were more likely to indicate that nobody should engage in actions evaluated as immoral relative to actions evaluated as personally negative; conversely, participants were more likely to indicate that everybody should engage in actions evaluated as moral relative to actions evaluated as personally positive. We also found an unexpected three-way interaction with block, *F*(3, 195) = 2.66, *p* = .05, indicating that this interaction was strongest during the first two blocks. However, this effect was not replicated in the other experiments. In sum, these results are consistent with the general hypothesis that moral evaluations are associated with universality judgments to a greater degree than pragmatic evaluations.

#### Moral evaluations are extreme

Following the results of Experiment 1, we predicted that moral evaluations would be more extreme than pragmatic evaluations of the same actions, but not more positive or negative. Consistent with Experiment 1, we found no difference on the overall rated valence of actions when participants made moral (M = 4.21) or pragmatic (M = 4.27) evaluations (*p* = .64). As predicted, participants made more extreme moral (M = 5.53) than pragmatic (M = 5.32) ratings of the same actions, *F*(1, 65) = 3.93, *p* = .05 (see Table 2). Consistent with the prediction that participants would be able to switch between moral and pragmatic evaluative modes, the effect of evaluation type was not moderated by block (*p* = .35). These results indicate that moral evaluations were more extreme than pragmatic evaluations of the same actions.

**Table 2:** Mean responses following moral versus pragmatic evaluations in Experiment 2.

DV	Task	Block 1	Block 2	Block 3	Block 4	Average
Evaluation RT	Moral	3,961	3,232	2,945	2,614	3,188 (110)
	Pragmatic	4,180	3,429	3,098	2,838	3,386 (110)
Extremity	Moral	5.84	5.41	5.48	5.39	5.53 (.17)
	Pragmatic	5.39	5.18	5.37	5.35	5.32 (.17)
Universality RT	Moral	2,187	1,388	1,182	1,029	1,446 (87)
	Pragmatic	2,282	1,540	1,304	1,094	1,555 (87)

Means are provided for raw reaction times (RT; in milliseconds) and extremity of responses for each block. Excludes all trials with reaction times >10,000 ms. Overall scores may not reflect mean Block scores due to rounding errors and missing trials. Pooled standard errors in parentheses.

#### Moral evaluations are fast

Following the results of Experiment 1, we predicted that moral judgments would be faster than pragmatic evaluations of the same actions. To test this hypothesis, we compared the reaction times of moral versus pragmatic evaluations. As predicted, participants were faster to provide moral (M = 3,188 ms) than pragmatic (M = 3,386 ms) evaluations of the same actions, *F*(1, 65) = 14.18, *p*<.01. Participants were also faster to respond in later blocks, *F*(3, 195) = 85.85, *p*<.01, indicating a task-learning effect; however, the effect of condition was not moderated by block (*p* = .94). Replicating the results from Experiment 1, moral evaluations were faster than pragmatic evaluations across the blocks, suggesting that participants were able to switch back and forth between moral and pragmatic evaluative modes.

### Discussion

The results of the first two experiments provide convergent evidence that thinking morally is associated with universality. Specifically, participants were not only faster to make a universality judgment following a moral than a pragmatic evaluation, but mean moral judgments were more highly correlated with mean universality judgments than were pragmatic judgments. Comparing the effects of moral and pragmatic evaluation is important because it illustrates how easily people can depart from rational, pragmatic decision-making and shows that this departure has important implications for evaluative outcomes. However, since moral judgments were only compared to pragmatic evaluations, any inferences about the nature of moral evaluation from the first two experiments must rely on both the nature of pragmatic judgment and the nature of the psychological contrast between moral and pragmatic construal (e.g., moral judgments may be less complex).

To address these concerns, we compared moral judgment to an alternative type of judgment in experiment 3—a simple judgment about whether each action is pleasant or unpleasant [65]. We also reasoned that the hedonic evaluations were likely to be highly subjective, which might lead to relatively weak associations with universality, even relative to pragmatic evaluations. The outcomes of moral evaluation were thus compared with the outcomes of a simple hedonic evaluation. We also compared differences between moral and hedonic evaluation with differences between moral and pragmatic evaluation to see if the non-moral condition (pragmatic versus hedonic) had any major implications for interpreting the results from the first two experiments.

The results from the first two experiments indicated that participants were able to shift back-and-forth between moral and pragmatic evaluations with distinct consequences, indicating that the evaluations are sensitive to construal. Participants were able to evaluate a series of actions using moral considerations and then quickly shift to evaluate a separate series of actions using pragmatic considerations. Although this level of flexibility is impressive, no single participant was forced to provide moral *and* pragmatic evaluations of the same object(s). If moral evaluation is truly flexible, participants may be able to evaluate the exact *same action* in very different ways depending on their current evaluative mode. Moreover, this flexibility should lead to different evaluative outcomes for the same stimuli even when the different evaluations take place only moments apart. For example, a person who is considering the pragmatic costs of recycling but is suddenly reminded to consider its moral implications may have a sudden change of heart about discarding an empty bottle in the trash. Although this example seems intuitively plausible, human concerns about being and appearing consistent [66], [67], along with psychological anchoring processes [68] render this a conservative test of the flexibility hypothesis. In Experiment 3, each participant made both moral and non-moral evaluations of the same set of actions during the same experimental session, and type of evaluation switched semi-randomly on a trial-by-trial basis (see below). We predicted that moral evaluations would be associated with different evaluative outcomes (e.g., universality) relative to non-moral evaluations, even when participants made both forms of evaluation toward the same objects during the same session.

## Experiment 3

### Overview and Predictions

We have presented evidence that participants were able to shift back-and-forth between moral and non-moral evaluative modes in a relatively flexible fashion. The results from the first two experiments suggest that people can shift between moral and non-moral evaluative modes in a *tonic* fashion—shifting modes for a series of trials (blocks) at a time. A stronger form of our dynamical systems approach would predict that people can shift between moral and non-moral evaluative modes in a *phasic* fashion. If so, it would suggest that the construal process can influence on evaluation on a moment to moment basis. In Experiment 3, participants switched semi-randomly between moral and non-moral (pragmatic or pleasantness) evaluations on a trial-by-trial basis.

As in the first two experiments, participants rated whether each action was moral or non-moral (pragmatic or pleasant) and then rated whether the action should be universally prohibited/required. However, in this experiment we compared differences between moral and pragmatic evaluations with differences between moral and hedonic evaluations to see if the non-moral condition (pragmatic versus hedonic) had any major implications for interpreting the results from the first two experiments. This allowed us to determine whether moral evaluations were more strongly associated with universal prescriptions than two forms of non-moral evaluation. We predicted that moral evaluations would be associated with different evaluative outcomes (e.g., universality) relative to non-moral evaluations, even when participants were forced to shift back and forth between moral and non-moral evaluations every few seconds.

### Material and Methods

#### Participants

One hundred and forty-eight undergraduate psychology students (84 females; mean age = 20) participated for partial course credit. Three participants did not complete the experiment and were not included in the analysis.

#### Procedure

The procedure was similar to the previous experiment, with three important differences. First, participants were randomly assigned to make non-moral evaluations on the basis of pragmatic or hedonic concerns. Thus, half the participants made moral and pragmatic evaluations (as in the previous two experiments), and half the participants made moral and hedonic evaluations. Second, participants shifted between moral and non-moral evaluations on a trial-by-trial basis. However, the order of every pair of moral and non-moral trials was randomized to ensure that participants could not anticipate that every even (or odd) numbered trial was always moral (non-moral). For example, if participants made a moral and then a non-moral evaluation on the first two trials, the order of the moral and non-moral evaluations in the subsequent two trials was randomly determined. This design allowed us to test whether the previous effects of moral versus non-moral evaluations were based on some kind of tonic moral versus non-moral mindset, or whether participants were capable of flexibly shifting from moral to non-moral evaluations in a flexible and rapid (phasic) fashion. Third, each participant made both moral and non-moral evaluations of the same set of actions during the same experimental session (in the previous experiments, participants never evaluated the same action twice).

#### Analyses

To assess differences between moral versus pragmatic evaluation, we conducted 2 (evaluation type: moral, non-moral)×2 (non-moral: pragmatic, hedonic) repeated-measures analyses on the speed with which participants made evaluations, the overall valence and extremity of their evaluations, as well as their reaction times to subsequent universality ratings. To analyze reaction times, we removed trials with extremely long (>10,000 ms) reaction times and log-transformed all remaining reaction times. To ease interpretation, all reported means are based on raw reaction times.

### Results

#### Moral evaluations are associated with universality

Our primary prediction in Experiment 3 was that moral evaluations would be more strongly associated with universality judgments than non-moral evaluations, regardless of whether moral evaluations were contrasted with pragmatic or hedonic evaluations. To test this hypothesis, we compared the reaction times of universality judgments following moral versus non-moral (pragmatic and hedonic) evaluations. As predicted, participants were faster to make universality judgments following moral (M = 1,651 ms) compared to non-moral (M = 1,701 ms) ratings, *F*(1, 143) = 10.81, *p*<.01. There was no main effect of the non-moral control condition, *F*(1, 16848) = 1.47, *p* = .23, and the effect of evaluation type did not differ when compared with pragmatic versus hedonic evaluations (*p* = .91). Estimated G matrix was not positive definite during the analysis of cross-level interactions when the between-subjects variables were modeled as random effects. Therefore, between-subjects main effects and interactions were modeled as fixed effects. The degrees of freedom reflect the difference between random and fixed effects parameters. Replicating and extending the results from the first two experiments, moral evaluations facilitated universality judgments more than pragmatic or hedonic evaluations throughout the session, suggesting that participants were able to switch between moral and non-moral modes of evaluation. More importantly, Experiment 3 provided evidence that construal affected universality judgments for the same actions within subjects, such that evaluating the same people responded differently when evaluating the same action morally versus non-morally.

Following the results from the first two experiments, we predicted that universality judgments would be more highly correlated with preceding moral than non-moral (pragmatic or hedonic) evaluations. As predicted, a two-way interaction between evaluation type and the preceding moral/non-moral rating, *F*(1, 143) = 202.39, *p*<.01, indicated that participants' universality ratings were more strongly associated with preceding moral (ß = .72) than non-moral (ß = .58) ratings. As such, participants were more likely to indicate that nobody should engage in actions evaluated as immoral relative to actions evaluated as pragmatically or hedonically negative; conversely, participants were more likely to indicate that everybody should engage in actions evaluated as moral relative to actions evaluated as pragmatically or hedonically positive.

These effects were qualified by a three-way interaction between evaluation type, and whether the non-moral condition was pragmatic or hedonic, *F*(1, 16,844) = 19.15, *p*<.01. When the control condition involved pragmatic evaluation, there was a two-way interaction between evaluation type and the preceding moral/pragmatic rating, *F*(1, 73) = 53.13, *p*<.01, indicating that participants' universality ratings were more strongly associated with preceding moral (ß = .72) than non-moral (ß = .61) ratings. However, when the control condition involved hedonic evaluation, the two-way interaction between evaluation type and the preceding moral/hedonic rating was stronger, *F*(1, 70) = 159.98, *p*<.01, indicating that participants' universality ratings were more strongly associated with preceding moral (ß = .72) than non-moral (ß = .56) ratings, and that this difference was greater than in the moral/pragmatic condition. Although moral evaluations were strongly linked to universality judgments of the same action in both conditions, these results suggest that participants may have been more willing to generalize their pragmatic evaluations to others than their hedonic evaluations. However, the most robust effect remains that universality judgments (universally prohibited/required) were more highly correlated with moral than non-moral ratings—whether they were pragmatic or hedonic in nature. These results are consistent with the general hypothesis that moral evaluations are associated with universality judgments to a greater degree than other forms of evaluation.

#### Moral evaluations are extreme

Following the results of the first two experiments, we predicted that moral evaluations would be more extreme than non-moral evaluations of the same actions, but not more positive or negative. Consistent with the previous experiments, we found no difference on the overall valence ratings of actions when participants made moral (M = 4.19) or non-moral (M = 4.17) evaluations, *F*(1, 143) = .21, *p* = .65. As predicted, participants made more extreme moral (M = 5.10) than non-moral (M = 4.64) ratings of the same actions, *F*(1, 143) = 26.67, *p*<.01 (see Table 3). There was no effect of non-moral (pragmatic versus hedonic) evaluation (*p* = .60), and the effect of evaluation type was not moderated by the non-moral evaluation (*p* = .66). In other words, the nature of the non-moral condition did not make a difference: people's moral evaluations were more extreme than their pragmatic *or* hedonic evaluations of the same actions.

**Table 3:** Mean responses following moral versus pragmatic and moral versus hedonic evaluations in Experiment 3.

DV	Task	Moral versus Pragmatic	Moral versus Hedonic
Evaluation RT	Moral	3,656 (102)	3,667 (105)
	Non-moral	3,857 (102)	3,787 (105)
Extremity	Moral	5.07 (.17)	5.14 (.17)
	Non-moral	4.56 (.17)	4.71 (.17)
Universality RT	Moral	1,569 (76)	1,733 (78)
	Non-moral	1,605 (76)	1,798 (78)

Means are provided for raw reaction times (RT; in milliseconds) and extremity of responses for each block. Excludes all trials with reaction times >10,000 ms. Overall scores may not reflect mean Block scores due to rounding errors and missing trials. Pooled standard errors in parentheses.

#### Moral evaluations are fast

Following the results of the first two experiments, we predicted that moral judgments would be faster than non-moral evaluations of the same actions. To test this hypothesis, we compared the reaction times of moral versus pragmatic and hedonic evaluations. As predicted, participants were faster to provide moral (M = 3,661 ms) than non-moral (M = 3,822 ms) evaluations of the same action*s*, *F*(1, 143) = 28.86, *p*<.01 (see Table 3). This increase in overall reaction time relatively to the previous two experiments is likely due to the fact that participants were forced to switch evaluations on a trial-by-trial basis in Experiment 3, inducing a task-switching cost [69]. There was no effect of the non-moral (pragmatic versus hedonic) evaluation (*p* = .72), and the effect of evaluation type was not moderated by the non-moral evaluation (*p* = .15). In other words, the nature of the non-moral condition did not make a difference: people were faster to make moral evaluations than pragmatic *or* hedonic evaluations. Replicating and extending the results from the first two experiments, moral evaluations were faster than non-moral evaluations throughout the study, suggesting that participants were able to switch back and forth between moral and non-moral evaluative modes on a trial-by-trial basis.

### Discussion

The results from Experiment 3 replicate and extend the results from the first two experiments. The three experiments provide convergent evidence that thinking morally is associated with universality. Specifically, participants were not only faster to make a universality judgment following a moral than a non-moral evaluation, but mean moral judgments were more highly correlated with mean universality judgments than non-moral judgments. By replicating this pattern of effects when comparing moral with both pragmatic and hedonic modes of evaluation, we have increased confidence that the effects of moral evaluation are not merely a consequence of the psychological contrast between moral and pragmatic modes. However, the results of Experiment 3 indicated that different non-moral modes of evaluation are not equivalent: people seemed more willing to universalize their pragmatic than their hedonic evaluations.

Experiment 3 also provided the first evidence that people are able to shift back-and-forth between moral and non-moral evaluative modes in a highly flexible fashion—shifting construal on a trial-by-trial basis. Whereas the results from the first two experiments provided evidence that people could shift between moral and non-moral evaluative modes in a *tonic* fashion—shifting modes for a series or trials at a time—Experiment 3 indicated that people can shift on-line between moral and non-moral evaluative modes in a *phasic* fashion. This suggests that the effects of construal are not limited to evaluative modes or mindsets. Further, showing these differences *within* participants indicates that construal can override consistency motives [66], [67] and psychological anchoring [68].

## General Discussion

We present three experiments showing that moral evaluations are susceptible to top-down influences. Specifically, we show that people can deliberately construe a wide variety of actions through either a moral or a non-moral lens with different consequences for their evaluations. Thus, moral evaluation is not strictly a bottom-up process. The current research provides evidence that moral and non-moral construals of the same actions lead to distinct evaluative outcomes. Specifically, the moral evaluative mode elicited faster, more extreme and more universally prescriptive evaluations than non-moral evaluative modes, consistent with longstanding assumptions about morality. In short, evaluating an action in moral terms increased people's inclination to render judgments in absolutes—more simple, extreme, black-and-white evaluations. These differences in evaluative outcomes are consistent with the contention that moral and non-moral construals triggered different evaluations. In addition, our experiments suggest that people can shift back-and-forth between moral and non-moral evaluations of the same actions very quickly, consistent with dynamical models of evaluation [24], [28], [29].

Much of the previous research on morality has made an implicit assumption that moralization leads people to reflexively construe certain actions or dilemmas as moral. Although this may certainly be the case for many issues, such as murder and incest, the current research suggests that people can construe and evaluate a host of issues according to moral standards [45]. Thus, while moralization involves the development of relatively stable moral contents (e.g., standards and values) and may instigate the construal of certain acts in moral terms, whether these contents influence an evaluation at any given moment likely depends on whether an action or issue is construed in moral or non-moral terms. As such, it seems likely that issues that have not necessarily been extensively moralized (e.g., recycling) may allow for the most flexible evaluations and lead to the largest differences between moral and non-moral evaluative modes [70]. In contrast, actions that are highly moralized (smothering a baby) or mundane (wearing a sweater vest) may allow for less flexibility [23].

To investigate the influence of construal, we instructed participants to evaluate the same stimuli in moral versus non-moral (e.g., pragmatic) terms. Our experimental paradigm—which holds stimuli constant while varying the mode of evaluation—allowed us to investigate how flexibly moral versus non-moral evaluative modes can be applied to judgment of the same stimuli and ensured that differences observed in the nature of evaluative outcomes were due to differences in the nature of evaluative construal rather than the stimuli. As we predicted, evaluating actions on the basis of moral versus non-moral considerations lead to different evaluative outcomes. Specifically, the present data suggests that moral evaluations are more likely to be applied universally to others. In all three experiments, we found that moral evaluations were more strongly associated with universal prescriptions than non-moral evaluations. Future research should explore the relationship between moral evaluation and universality, including whether the effects of universality extend across time as well as people and the implications of these associations for human judgment and decision-making.

Building on our dynamical model of the evaluative system, we distinguish between the *contents* (e.g., attitudes and standards) and *processes* (e.g., mental operations and computations) of evaluation [24], [28], [29]. Accordingly, as long as there are some moral *contents* that participants can bring to bear on their evaluation, moral evaluations may be applied to actions that have not been typically seen as moral. For example, one can bring to mind the moral aspects of recycling (e.g., saving the environment) even if more pragmatic aspects normally predominate (e.g., the pain of driving to the local recycling depot). Thus, the current work extends the research by Skitka and colleagues [23] by showing that evaluating an issue as moral (or not) varies not only across individuals, but within individuals within seconds as a function of the construal the person is applying. Indeed, Experiment 3 provided evidence that construal influenced universality judgments for the same actions *within* the same people.

### Moral Construal

The current research manipulates the *construal* people use to evaluate different stimuli. Many others have proposed that moral cognition can be understood by the processes involved in moral reasoning rather than final judgments [14], [15]. Kohlberg [27] had participants respond to moral dilemmas and identified their stage of moral development on the basis of their reasoning. Rationalist approaches in moral psychology stress that moral judgments are reached through a process of reasoning and reflection [15], [16], [71]. More recently, researchers have challenged the view that moral reasoning is the sole or even primary means by which moral judgments are made, arguing that certain situations automatically elicit moral intuitions, which guide moral judgments [1]. According to the intuitionist model, moral reasoning frequently follows an initial judgment, providing a *post hoc* justification but not the causal impetus for a moral judgment. From the intuitionist perspective, unconscious, affective responses guide reactions to these morally charged scenarios and people often engage in deliberate reasoning only after they have already made an initial moral judgment.

The two stage models of ethical decision-making argue that the “eliciting situation” (e.g., a stimulus, situation or course of action) is only likely to be judged as morally right or wrong when prior processes first determine that the situation is to be evaluated in moral terms. Given the variety of actions that elicited differences between moral and non-moral evaluations in the current experiments, we contend that moral evaluation can extend beyond the actions and dilemmas that are typically examined in studies on moral cognition. Thus, while certain eliciting situations, such as smothering a baby [72], may serve to directly trigger moral awareness in addition to providing a basis for the resultant moral judgment, many situations are highly sensitive to framing and construal [73]. For instance, research suggests that people can make decisions using different perspectives, from the legal viewpoint of a judge to the moral viewpoint of a citizen, and these different perspectives can shape the processes underlying legal and moral decisions [46].

Although the cognitive reasoning or intuitionist models of moral evaluation are not necessarily inconsistent with a dissociation between awareness and judgment stages in moral evaluation, by using highly moralized stimuli and/or by asking people to form moral judgments (cuing moral awareness), these research traditions may over-estimate the extent to which moral evaluation is automatically triggered by stimulus features. Paradigms designed to examine moral judgment in both the moral reasoning and intuitionist traditions are predominantly stimulus-driven, confronting participants with situations or dilemmas that are assumed *a priori* to be morally relevant (or not). Many of these studies cannot easily discriminate effects due to differences between moral and non-moral forms of evaluation from effects due to stimulus differences; even in studies that contrast judgments and decisions made in response to ostensibly moral and non-moral situations, these conditions differ both in type of evaluation and type of stimuli. We therefore suggest that extant research on the psychological underpinnings of moral evaluation does not provide much direct evidence that moral awareness (choosing to evaluate stimuli in moral terms) is independent of moral judgment. Experimental approaches like the one employed here are important for several reasons. First, they allow for a test of the contention that many of the same actions can be evaluated in moral and non-moral ways and that these different types of evaluation have distinct evaluative outcomes. Second, this approach helps disentangle the awareness and judgment stages of moral evaluation. By having participants evaluate the same actions in moral and/or non-moral terms we directly tested whether evaluating stimuli in moral terms gave rise to distinct outcomes. Research in the cognitive reasoning tradition that explicitly directs participants to evaluate situations such as the Heinz Dilemma in moral terms lacks the non-moral control conditions necessary to dissociate these processes. In contrast, research in the intuitionist tradition, in which participants evaluate stimuli that are presumably moral (or not), cannot distinguish effects due to moral evaluative processes from effects due to stimulus characteristics. However, if different evaluative outcomes are observed when participants evaluate the same actions in moral versus non-moral terms, this supports the notion that moral processes themselves have evaluative consequences beyond the consequences associated with specific stimulus characteristics. By directly comparing the evaluative outcomes of moral versus non-moral modes of evaluation, we found that a moral evaluation elicited faster, more extreme and more universally prescriptive evaluations than non-moral evaluations.

We are not suggesting, however, that moral and non-moral (i.e., pragmatic or hedonic) modes of evaluation are completely independent: differences observed between moral and non-moral evaluation do not imply that the two forms of evaluation do not share many of the same underlying processes. Many neural component processes—especially those involved in representing value—are likely common to both forms of evaluation [36]. Further, moral and pragmatic evaluations of the same action may often lead to the same behavioral outcomes. Indeed, religious and secular institutions impose punishments on many forms of self-interested behavior to help ensure that pragmatic and moral concerns are closely aligned to the benefit of the collective. For example, the decision to commit a crime is not often only immoral, but is likely to incur severe legal punishments. In this way, legal and social sanctions act as deterrents for otherwise “immoral” behavior. Humans have spent centuries creating legal systems and social institutions (including religions) that align pragmatic rewards and punishments with moral concerns. This normally strong relationship between moral and non-moral evaluations mitigates potential differences, and makes our experimental tests of differences between these evaluative modes conservative.

### Lay Definitions of Morality

One of the major questions facing moral psychology is how one knows whether something is in fact a *moral* issue [74], [75], [76]. For the most part, researchers have used theoretical rationale or face validity as the primary criterion for morality, assuming that acts such as incest and murder are likely chronically construed as moral and that attributions of blameworthiness reflect moral evaluations. In the current research, we relied on participants' lay understanding of moral and non-moral evaluation. In some regards, this is a strength of the current research as it bypasses assumptions on the part of the researchers about the nature of moral versus non-moral modes of evaluation. It does, however, raise the possibility that the differences observed between moral and non-moral evaluation may have stemmed, at least in part, from participants' lay theories about the *difference* between these two dimensions of evaluation because our paradigm made participants aware that they were providing both moral and non-moral evaluations. However, a similar pattern of results holds for several non-moral evaluations (pragmatic and hedonic), suggesting that our effects are not specific to lay theories about the distinction between moral and pragmatic evaluations. In any event, future research should examine whether making this contrast salient enhances the reported differences.

### Future Research

In each of the experiments reported above, we instructed people to evaluate the same stimuli in moral versus non-moral terms. This experimental approach, which holds stimuli constant while varying the mode of evaluation, is important because it allows us to investigate how construal processes can be applied to judgment of the same stimuli, and because it ensures that differences observed in the nature of evaluative outcomes are due to differences in the nature of evaluative *processing* rather than the *stimuli*. As we noted above, we intentionally used the term “processed” in the broad sense to include stimulus construal. Although our experimental design ensured that participants evaluated the exact same stimuli in moral and non-moral evaluative modes, this does not preclude the possibility that different underlying representations (i.e., contents) were activated and applied to the evaluations in both modes. We hold open the possibility that any differences in evaluative outcomes may reflect different underlying representations. For example, evaluating the moral implications of recycling may activate a different set of contents (e.g., representations based on beliefs and attitudes about global warming, social responsibility, etc.) than evaluating the pragmatic implications of recycling (e.g., the costs and benefits in terms of the time and money involved in recycling). Future research should use a combination of behavioral and physiological measures to assess underlying differences in process versus content [77].

Similarly, neuroimaging could be used to help understand the hierarchical relationship between the brain systems implicated in moral construal and evaluation, since these systems are frequently confounded in extant research. We expect that the region of ventral medial prefrontal cortex frequently implicated in moral decision-making studies [78], [79] may be sensitive to top-down construals instigated by higher-order control processes implemented by the fronto-parietal network [28], [80], [81]. This work may also elucidate the mental computations that underlie moral and non-moral evaluation.

Evidence that moral versus non-moral evaluations can be moderated by construal, applied to wide range of actions, and are associated with distinct evaluative outcomes, has a number of important implications. First, the processes associated with morality may be sensitive to motivation and social context. Moral framing has been shown to increase generosity in economic games [82]. Likewise, people primed with religious constructs may be more likely to see the moral implications of their actions, leading to more generous behavior [83]. Framing issues in terms of their moral implications may also reduce selfish behavior in a variety of contexts, such as cheating or paying taxes. Second, our data suggest that morality is not always associated with specific issues, but stems from the construal of those issues. Third, it raises the possibility that moral construal may lead to systematic biases in decision-making and behavior. For example, considering the pragmatic versus moral implications of voting might have a profound effect on voting behavior. If people focus on the time and energy involved, they may be unlikely to vote; alternatively, if the same people focus on their moral duty as voters for preserving a healthy democracy, they may be willing to vote despite the personal costs. As such, construing the same action in moral versus pragmatic terms may ultimately lead to different evaluations and behavior [84], [85].

### Conclusion

People engage in countless actions on a daily basis and these actions can be based on a number of considerations, from gut instinct to a rational cost-benefit analysis. The current research suggests that people can also base their actions on their moral standards, and using these standards alters the mental operations used to evaluate those actions. As a consequence, ostensibly moral acts may be construed and processed according to other standards, and vice-versa. The effects of construal highlighted in the current research suggest that generating an appropriate construal (moral or otherwise) may be one of the most important aspects of moral or ethical decision-making [86]. The failure, for example, to consider the pragmatic implications of certain decisions could lead to unnecessarily swift or extreme decisions. Conversely, the failure to consider the moral implications of one's actions may ultimately lead people to act immorally in pursuit of pragmatic ends [87]. Future research should continue to investigate why people evaluate certain actions in moral terms as opposed to analyzing their pros and cons or considering their hedonic value.

## Supporting Information

Appendix S1 (A) Participants were presented with 104 actions one at a time on a desktop computer using E-Prime (Experiment 1). (B) Participants were presented with an additional 16 additional actions, for a total of 120 actions (Experiments 2 and 3).
(DOCX)
