# Supplementary material for: The Importance of Moral Construal: Moral versus Non-Moral Construal Elicits Faster, More Extreme, Universal Evaluations of the Same Actions
Source: PLoS One. 2012 Nov 28;7(11):e48693. doi: 10.1371/journal.pone.0048693 (PMC3509100; doi:10.1371/journal.pone.0048693)
Supplement: Appendix S1 — (A) Participants were presented with 104 actions one at a time on a desktop computer using E-Prime (Experiment 1). (B) Participants were presented with an additional 16 additional actions, for a total of 120 actions (Experiments 2 and 3). (DOCX) [file pone.0048693.s001.docx]

**Appendix S1 A**

ride a bike rather than

drive a car

study

save for retirement

work hard

turn off the lights

be punctual

conserve water

get married

carpool

ride the bus rather than

drive a car

recycle

obey traffic lights

listen to parents

support friends

be honest

help an older person cross

the road

report a crime

treat a friend to dinner

plant a tree

exercise regularly

eat healthily

be loyal to a friend

signal when changing

lanes

obey stop signs

be loyal to a romantic

partner

meditate

adopt a child

confront a bully

donate to charity

help a friend with

homework

pay parking tickets

vote

buy birthday gifts for

friends

return a lost wallet

walk a long way to work

donate a kidney

put money in someone's

expired parking meter

give change to the

homeless

volunteer in a hospital

volunteer at a shelter

pay taxes

confess to a crime

confront a friend about a

drug problem

date a coworker

shovel a neighbor’s walk

take in a homeless person

admit to a lie

rescue someone from a fire

rescue someone from a car accident

express an unpopular

opinion

buy organic food

challenge a friend's racist

remark

park in a handicap spot

gossip about friends

shoplift

make an illegal U-turn

cheat on a test

cut into line

plagiarize parts of a paper

litter

skip class

drive too fast

lie to get a free meal

keep a lost wallet

keep a lost IPod

eat fast-food

buy an SUV

cheat on a tax return

leave a meal unfinished

leave a restaurant without

paying

double park

flatter a boss with a lie

steal supplies from work

tell a white lie

spread a false rumor

eat too much

drink too much

throw away left-overs

drive drunk

cheat on a romantic partner

murder a family member

have unsafe sex

gamble

pick on someone with a

disability

bully another person

sexually harass a coworker

lie to get a job

lie on a college application

kidnap a child

carry a concealed knife

carry a concealed gun

beat up a friend

emotionally abuse a

romantic partner

emotionally abuse a child

buy alcohol for minors

buy cigarettes for minors

haze new students

tell a child they are fat

vandalize a park

vandalize an office

building

steal a car

rob a store

join a riot

knowingly spread an STD

**Appendix S1 B**

write an email

take notes on a lecture

watch a sitcom

drink from a water-fountain

go to the movies

ride the elevator

take a walk in the park

sit by a pond

admire the view

drink a glass of water

cook dinner

eat a sandwich

read a novel

surf the internet

get a haircut

wear a sweater-vest
